# Supplementary material for: PI3K/mTOR Inhibitor Induces Context-Dependent Apoptosis and Methuosis in Cancer Cells
Source: Pharmaceuticals (Basel). 2025 Dec 4;18(12):1849. doi: 10.3390/ph18121849 (PMC12736009; doi:10.3390/ph18121849)
Supplement: Supplementary file 1 [file pharmaceuticals-18-01849-s001.zip › pharmaceuticals-3991974-supplementary.pdf]

Supporting Information

PI3K/mTOR Inhibitor Induces Context-Dependent Apoptosis and Methuosis in Cancer Cells

Xiaoyuan Hua [1,†], Panpan Chen [1,†], Wanjing Zeng [1], Yuqiao Han [1], Yanzhi Guo [1], Yanmei Chen [1], Chuchu Li [1], Yijie Du [2,*], Mingliang Ma [1,3,*] and Suzhen Dong [1,*]

Table of Contents

1. **Supplementary figure S1: S3**
2. **Supplementary figure S2: S4**
3. **Supplementary figure S3: S5**
4. **Supplementary figure S4: S7**
5. **Supplementary figure S5: S8**
6. **Supplementary figure S6: S9**
7. **Supplementary figure S7: S10**
8. **Supplementary figure S8: S11**
9. **Supplementary figure S9 S12**

Supplementary figure S1:

Morphological changes of different tumor cell lines after administration of compound YYN-37 at concentration gradients for 24 hours. Scale bar =25 μm.

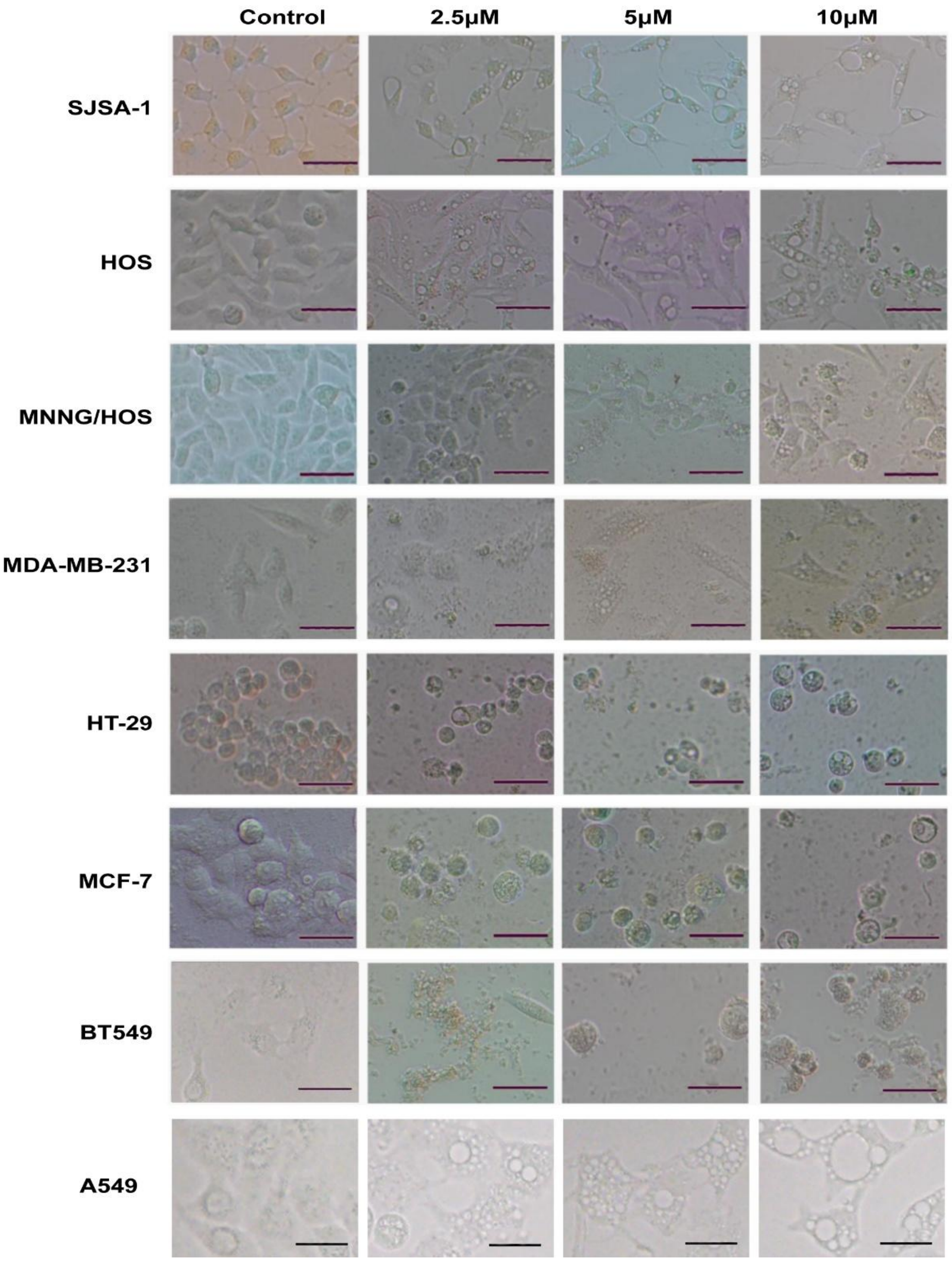

Supplementary figure S2:

Electron microscopy was used to detect the morphological changes of SJSA-1 cells induced by the compound YYN-37.

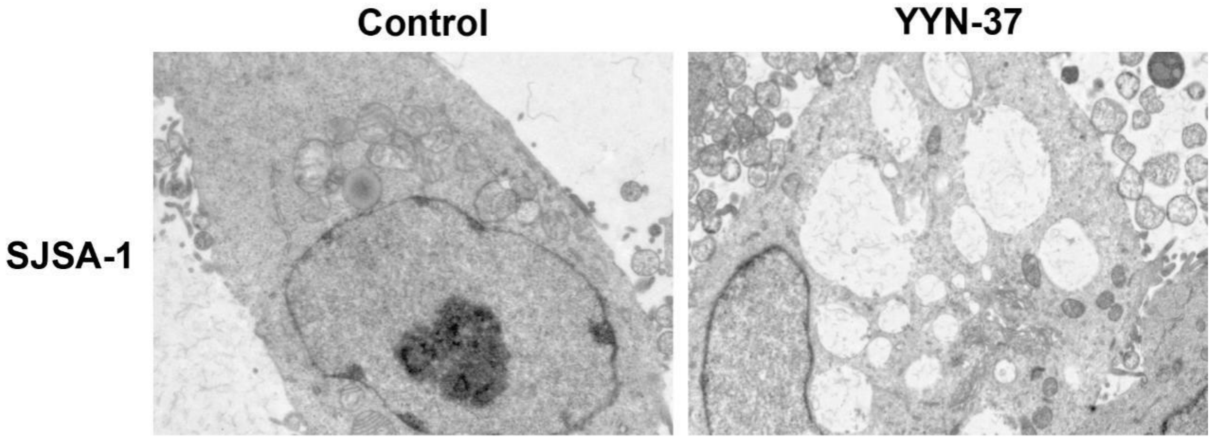

Supplementary figure S3:

PI3K III (Vps34) is involved in the cellular vacuolization induced by YYN-37. The AO fluorescence distribution in SJSA-1 cells treated with control cells (A), 2 μM LEU (B), 5 μM YYN-37 (C), 0.074 μM LEU + 5 μM YYN-37 (D), 0.22 μM LEU + 5 μM YYN-37 (E), 0.67 μM LEU + 5 μM YYN-37 (F), and 2 μM LEU + 5 μM YYN-37 (G). (H) Statistical analysis of the ratio of the red fluorescence area to the green fluorescence area. LEU represents leucine. N = 3. Scale bar =25 μm. One-way ANOVA statistical analysis was performed. Compared with the control group, *** indicates P < 0.001, and **** indicates P < 0.0001.

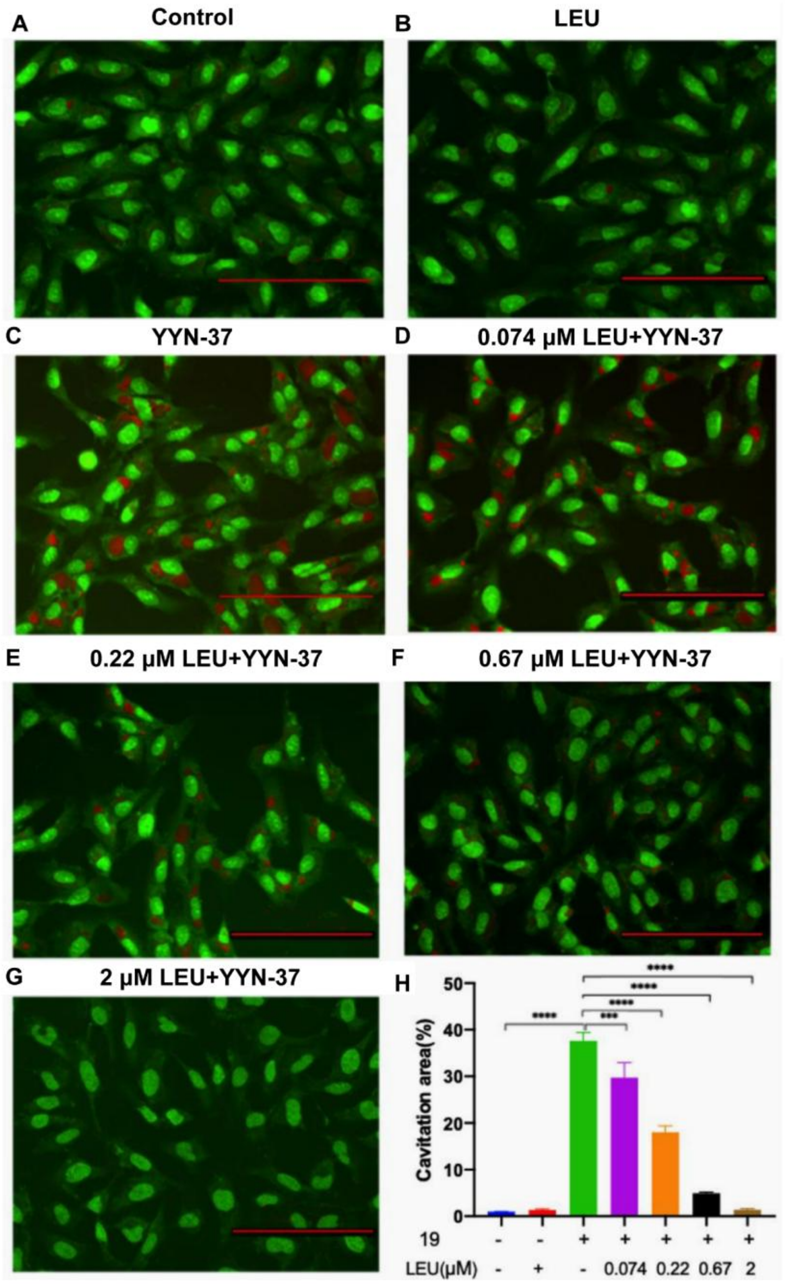

Supplementary figure S4:

A549 cells were transfected with VPS34 siRNA or scrambled siRNA. Then VPS34 expression was analyzed by western blotting (**A**). The morphology of the cells was shown in (**B**).

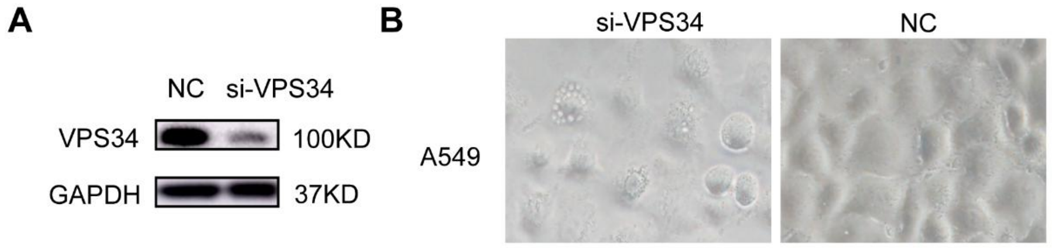

Supplementary figure S5:

GO enrichment analysis diagram of HCT-116 cells after YYN37 treatment compared with the control group

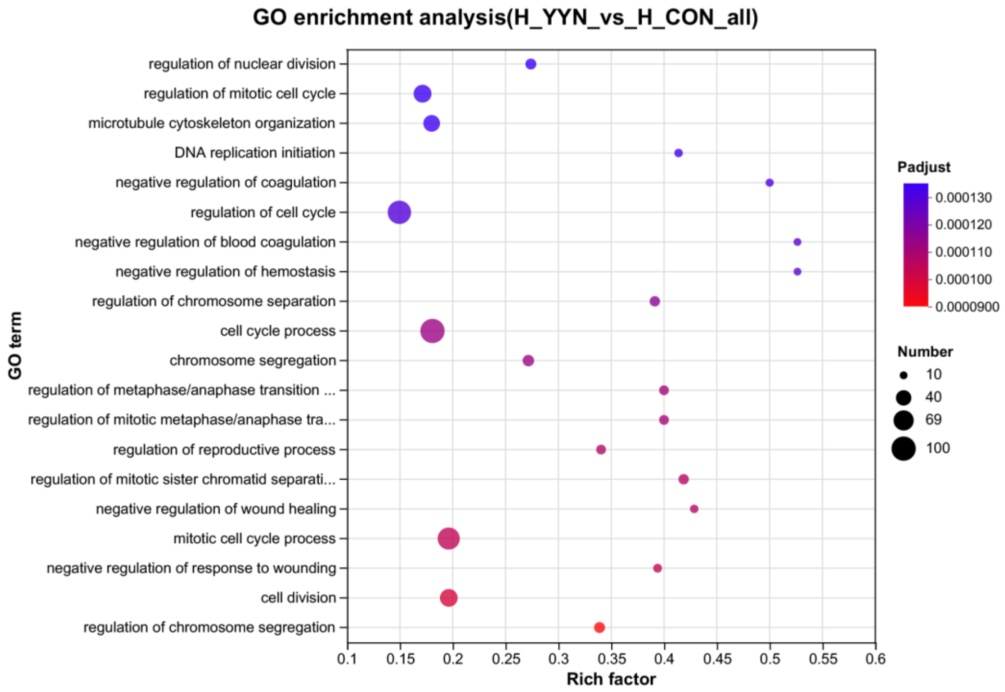

Supplementary figure S6:

The structure of YYN-37

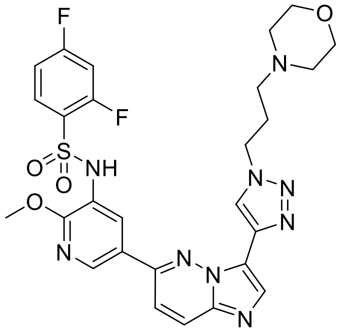

YYN-37

YYN-37: Yellow solid (yield: 63%); Melting point: 129.9-134.2 °C.^{1}H NMR (400 MHz, $CDCl_3$) δ 8.76 (s, 1H), 8.71 (s, 1H), 8.53 (s, 1H), 8.45 (s, 1H), 8.12 (d, J = 9.4 Hz, 1H), 7.82 (dd, J = 14.5, 7.8 Hz, 1H), 7.51 (d, J = 9.4 Hz, 1H), 7.01 – 6.86 (m, 2H), 4.66 (t, J = 6.8 Hz, 2H), 4.03 (s, 3H), 3.72 – 3.58 (m, 4H), 2.50 – 2.35 (m, 6H), 2.29 – 2.17 (m, 2H). ^{13}C NMR (101 MHz, $CDCl_3$) δ 166.2 (dd, J = 259.2, 11.6 Hz), 159.9 (dd, J = 259.2, 12.8 Hz), 155.7, 148.5, 140.5, 138.8, 136.8, 132.9, 132.1 (d, J = 10.7 Hz), 126.3, 125.9, 125.4, 123.4(dd, J = 13.7, 3.7 Hz), 121.8, 121.2, 121.1, 113.7, 112.2 (dd, J = 22.1, 3.5 Hz), 106.0 (t, J = 25.4 Hz), 66.9 (2C), 55.4, 54.5, 53.5 (2C), 48.7, 27.3. HRMS (ESI) m/z calcd for $C_{27}H_{27}F_2N_9O_4S$ $(M+H)^+$ 612.1953; found, 612.1967. Purity: 99.04%.

Supplementary figure S7:

NMR Spectra of YYN-37

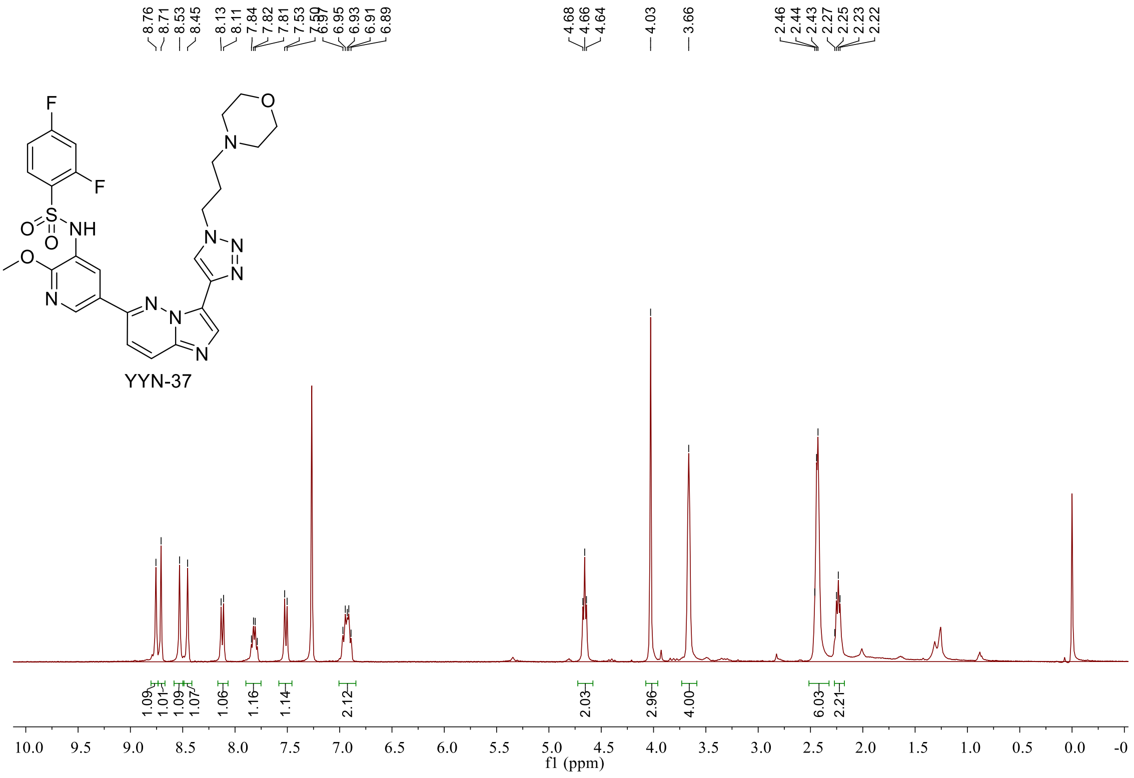

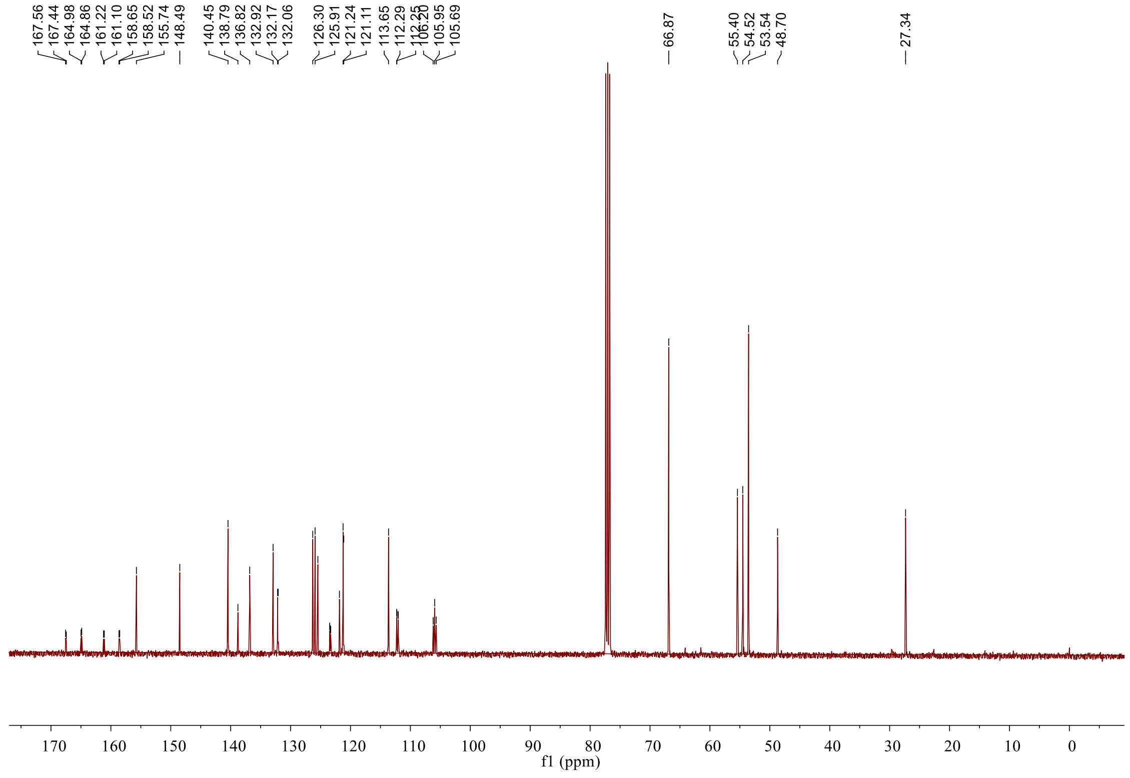

Supplementary figure S8:

HRMS Spectra of YYN-37

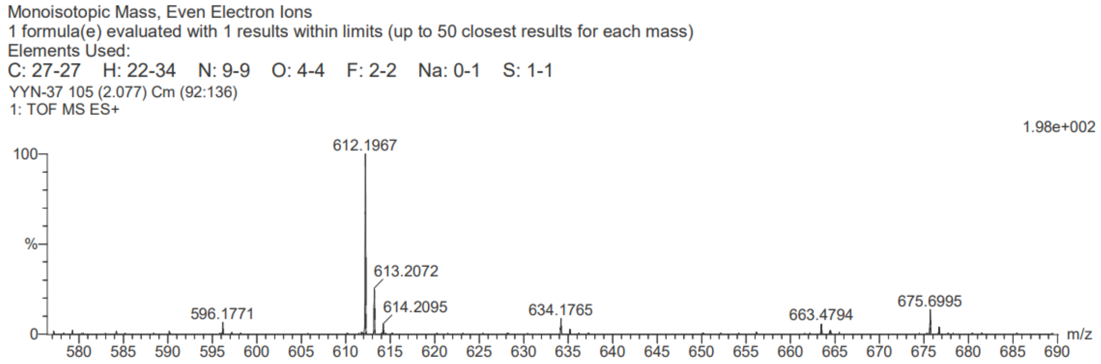

Minimum:				-1.5	
Maximum:		500.0	10.0	50.0	
Mass	Calc. Mass	mDa	PPM	DBE	Formula
612.1967	612.1953	1.4	2.3	17.5	C27 H28 N9 O4 F2 S

Supplementary figure S9:

HPLC Chromatograms of YYN-37

The purities of YYN-37 (>95%) were determined by HPLC with acetonitrile/buffer (1‰ HCOOH in water) as the mobile phase obtained on Thermo Scientific UltiMate 3000 HPLC.

HPLC conditions:

Column: Syncronis C18（4.6 × 250 mm, 5 μm）/ InertSustain C18（4.6 × 250 mm, 5 μm）

Wave length: 254 nm

Injection: 10 μL

The elution gradient is as follows:

Time	Flow (mL/min)	1‰ HCOOH in water	Acetonitrile
0	1	95%	5%
2	1	95%	5%
17	1	5%	95%
29	1	5%	95%
29	1	95%	5%
30	1	95%	5%

Chromatogram

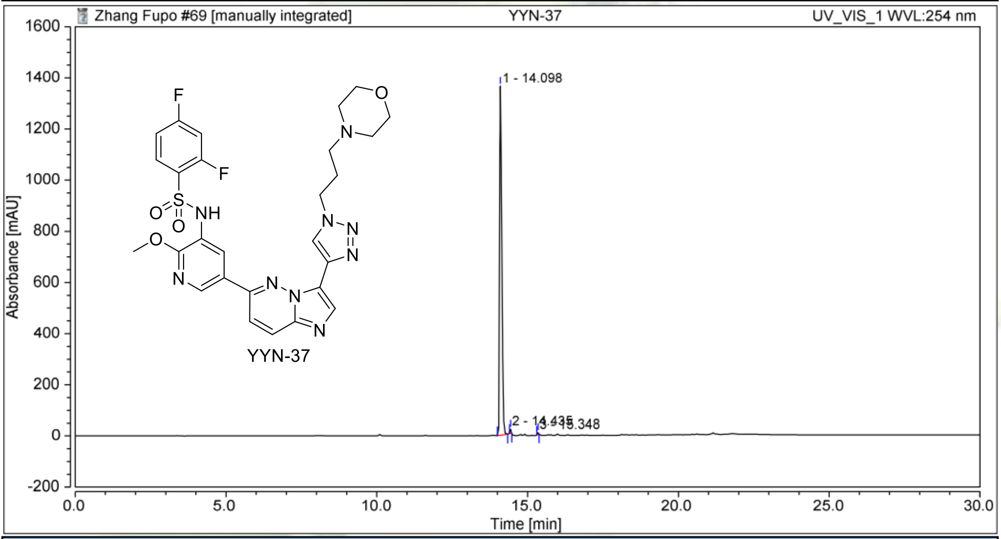

Integration Results

No.	Peak Name	Retention Time min	Area mAU*min	Height mAU	Relative Area %	Relative Height %	Amount n.a.
1		14.098	109.643	1364.613	99.04	98.23	n.a.
2		14.435	0.731	16.740	0.66	1.20	n.a.
3		15.348	0.336	7.880	0.30	0.57	n.a.
**Total:**			**110.710**	**1389.233**	**100.00**	**100.00**	
